# Altered spontaneous brain activity during dobutamine challenge in healthy young adults: A resting-state functional magnetic resonance imaging study

**DOI:** 10.3389/fnins.2022.1033569

**Published:** 2023-01-06

**Authors:** Yawen Liu, Haijun Niu, Tingting Zhang, Linkun Cai, Dong Liu, Erwei Zhao, Liang Zhu, PengGang Qiao, Wei Zheng, Pengling Ren, Zhenchang Wang

**Affiliations:** ^1^School of Biological Science and Medical Engineering, Beihang University, Beijing, China; ^2^Department of Radiology, Beijing Friendship Hospital, Capital Medical University, Beijing, China; ^3^Department of Ultrasound, Beijing Friendship Hospital, Capital Medical University, Beijing, China; ^4^National Space Science Center, Chinese Academy of Sciences (CAS), Beijing, China; ^5^National Research Center for Rehabilitation Technical Aids, Beijing, China

**Keywords:** rs-fMRI, ReHo, ALFF, dobutamine infusion, brain-heart interactions

## Abstract

**Introduction:**

There is a growing interest in exploring brain-heart interactions. However, few studies have investigated the brain-heart interactions in healthy populations, especially in healthy young adults. The aim of this study was to explore the association between cardiovascular and spontaneous brain activities during dobutamine infusion in healthy young adults.

**Methods:**

Forty-eight right-handed healthy participants (43 males and 5 females, range: 22–34 years) underwent vital signs monitoring, cognitive function assessment and brain MRI scans. Cardiovascular function was evaluated using blood pressure and heart rate, while two resting-state functional magnetic resonance imaging (rs-fMRI) methods—regional homogeneity (ReHo) and amplitude of low-frequency fluctuation (ALFF)—were used together to reflect the local neural activity of the brain. Logistic regression was used to model the association between brain and heart.

**Results:**

Results showed that blood pressure and heart rate significantly increased after dobutamine infusion, and the performance in brain functional activity was the decrease in ReHo in the left gyrus rectus and in ALFF in the left frontal superior orbital. The results of logistic regression showed that the difference of diastolic blood pressure (DBP) had significant positive relationship with the degree of change of ReHo, while the difference of systolic blood pressure (SBP) had significant negative impact on the degree of change in ALFF.

**Discussion:**

These findings suggest that the brain-heart interactions exist in healthy young adults under acute cardiovascular alterations, and more attention should be paid to blood pressure changes in young adults and assessment of frontal lobe function to provide them with more effective health protection management.

## 1. Introduction

Over the past years, the interactions between the central nervous system (brain) and the cardiovascular system (heart) have gained much attention and are becoming increasingly important as the underlying mechanisms of interactions between the two are better understood (Silvani et al., 2016; Greco et al., 2019; Vaccarino et al., 2021). Anatomically, the heart’s extensive efferent and afferent neural connections to the brain may be considered the physiological basis for the brain-to-heart pathway. The brain directly controls the heart through the sympathetic and parasympathetic nerves of the autonomic nervous system, which consists of polysynaptic pathways from cardiomyocytes back to peripheral ganglion neurons to central preganglionic and premotor neurons, the regulation of which involves complex cortical, subcortical, and medullary signaling (Pereira et al., 2013; Ritz et al., 2013; Silvani et al., 2016).

Both acute and chronic manifestations of imbalanced brain-heart interactions can negatively impact health. Recent studies have also shown that the neural correlates of cardiac regulation are broader than previously thought, and from a brain side, executive function and information processing speed, and to a lesser extent memory, are also associated with cardiovascular factors (Beissner et al., 2013; Hooghiemstra et al., 2019; Valenza et al., 2019). Previous studies in animal models and patients with heart failure have confirmed the important potential of brain-heart interactions for the treatment of cardiovascular disease (Choi et al., 2006; Vogels et al., 2007; Fan et al., 2021), the studies focus on patients with neurologically active diseases such as Alzheimer’s disease and dementia have shown that it is associated with changes in cardiac function (Oh et al., 2012; Cermakova et al., 2017; Nonogaki et al., 2017). More studies have shown that there is a certain relationship between the risk of developing heart failure and cognitive impairment and that this risk has a tendency to transfer to young people (Cermakova et al., 2017). Even in young people without cardiovascular disease, there is a link between subclinical cardiac dysfunction and subtle brain damage (Barasa et al., 2014; Schmidt et al., 2016; Lindgren et al., 2018). In this context, an in-depth understanding of the link between functional changes in the cardiovascular system and brain functional activity in young adults may provide valuable tools for early detection and treatment of pathological changes in brain-heart interactions. To our knowledge, there is currently a lack of studies on brain-heart interactions in healthy young adults.

Fluctuations in the indications of the cardiovascular system could be induced by different behavioral stimuli or under autonomic regulation (Harper et al., 2000). In contrast with the commonly used exercise stimulation and electrical stimulation, stress testing that exposes abnormal cardiac function is clinically performed by infusion of dobutamine (Casas et al., 2018). Dobutamine belongs to a class of drugs called inotropic agents, pharmacologically, dobutamine mainly stimulates vascular β1 receptors, elevating heart rate, and increasing myocardial contractility and cardiac output (Ruffolo, 1987; Tyrankiewicz et al., 2013; Guglin and Kaufman, 2014). Studies on the cardiovascular effects of dobutamine on healthy subjects have shown that changes in vital signs are linearly related to the infusion dose of dobutamine (Ahonen et al., 2008; Dubin et al., 2017). Tachycardia effects can occur after infusion rates above 2.5 μg/kg/min, possibly resulting in a marked increase in heart rate or blood pressure, especially systolic blood pressure. About 10% of patients in clinical studies had an increase in heart rate of 30 beats per minute or more, and about 7.5% had an increase in systolic blood pressure of 50 mmHg or more (Dubin et al., 2017; Krumpl et al., 2020).

Understanding the neural mechanisms underlying changes in the cardiovascular system requires simultaneous assessment of the activation of different brain regions to specific challenges. Electroencephalography (EEG) is the technique commonly used to investigate neural plasticity, offering advantages such as portability, ease of operation, and high temporal resolution. However, it gives poor spatial resolution and lacks functional brain area localization. In comparison, functional magnetic resonance imaging (fMRI), which utilizes blood oxygen level-dependent (BOLD) signals that respond to changes in neural activation, can non-invasively assess changes in activity in multiple brain sites in response to cardiac stress challenges (Kerem et al., 2021). In contrast to task-state fMRI, resting-state fMRI (rs-fMRI) is acquired in the resting state without stimulus or task. It focuses on fluctuation spontaneous BOLD signal changes that may be due to a variety of physiological phenomena such as blood pressure, cardiac and respiratory related rhythms, and thereby provides information on autoregulation mechanisms (Biswal et al., 1997; Dagli et al., 1999; Birn et al., 2006). Depending on the method of analysis, information about the function of specific brain regions (functional segregation) or the functional connectivity between different brain regions (functional integration) can be extracted (Tononi et al., 1994; Lv et al., 2018). Commonly used computational methods for functional integration include functional connectivity density analysis (FCD), ROI-based functional connectivity analysis, independent component analysis (ICA), and graph analysis (Biswal et al., 1995; Kiviniemi et al., 2003; Bullmore and Sporns, 2009; Tomasi and Volkow, 2010). Regional homogeneity (ReHo) and Amplitude of low frequency fluctuations (ALFF) are commonly used methods in the assessment of functional separation (Zang et al., 2004, 2007). Both ALFF and ReHo methods can be used to reveal local neural activity in the brain, and both methods are often applied together to reflect changes in spontaneous brain activity in different study subjects (Lin et al., 2017; Fang et al., 2022). In addition to the diversity of analytical methods, the development and application of analytical pipelines has made the processing of rs-fMRI much easier, leading to a wider application of rs-fMRI (Chao-Gan and Yu-Feng, 2010). As yet, however, there is only one study using dobutamine to induce changes in the cardiovascular system and observe its association with alterations in brain BOLD signal. In that study, cocaine-addicted subjects were recruited and subjected to different dobutamine infusion experiments (Liu et al., 2006). Although no positive results were obtained, it provided preliminary support for the view that can observe the brain-heart interactions through the dobutamine infusion challenge in the normal population.

In this study, we used rs-fMRI techniques to investigate the changes in spontaneous functional activates of the brain under changes in cardiovascular system function induced by dobutamine infusion and to further reveal the brain-heart interactions in healthy young adults. An expanded understanding of changes in brain function under dobutamine infusion is useful not only for understanding its basal effects in healthy young adults, but also for exploring its relationship with the brain to guide clinical application.

## 2. Materials and methods

### 2.1. Participants

An open volunteer recruitment was conducted through advertisements and fifty-two healthy volunteers were recruited in our study. The inclusion criteria were as follows: (1) age from 18 to 35 years; (2) right-handedness; and (3) no hypertension, hyperlipidemia, diabetes, or cardiovascular disease. The exclusion criteria were as follows: (1) Contraindications for MRI; (2) Plaques identified in carotid ultrasound; (3) Recent medication for problems such as antipsychotic, antidepressant or anxiety; and (4) presence of cardiac dysrhythmia through routine electrocardiogram (ECG) monitoring, and other medical conditions that make participants ineligible for dobutamine infusions. Participants were instructed to avoid exercise and consuming alcohol/caffeine ≥12 h prior to testing.

### 2.2. Data acquisition

**FIGURE 1:**
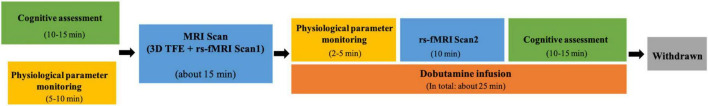
Data acquisition flow chart.

The data acquisition flow chart is shown in Figure 1.

Each participant underwent resting-state functional magnetic resonance imaging (rs-fMRI) scanning twice in a 3.0T Philips Ingenia scanner with a head-neck coil with a 16 channels head neck coil: normal resting state (Scan1) and dobutamine infusion state (Scan2). Each rs-fMRI scan lasted 10 min. Scan 1 was acquired before dobutamine infusion. Scan 2 was performed after 2 min of dobutamine infusion, as it has been shown that the effect of dobutamine on measured variables reached a steady state at 2 min and remained steady during the subsequent 10-min continuous infusion of the drug (Ogoh et al., 2017).

High-resolution T1-weighted images were collected using three-dimensional turbo field echo (3D-TFE) sequence (Hou et al., 2018; Bapst et al., 2020): TR = 10.3 ms, TE = 4.7 ms, FOV = 240 mm × 240 mm, Matrix = 220 × 219, slice thickness = 1.1 mm, number of slices = 200, Scan times = 3 min 26 s. The same rs-fMRI acquisition protocol was adopted for scan1 and scan2: TR = 3000 ms, TE = 35 ms, FOV = 240 mm × 240 mm, Matrix = 64 × 64, slice thickness = 4 mm, number of slices = 38, Scan times = 10 min 9 s. Participants were instructed to remain still, stay awake, and keep their eyes closed. After the scan we confirmed wakefulness by asking the participants.

The appropriate dose of dobutamine was configured before the experiment according to the participant’s weight. The rate of dobutamine infusion was 10–20 μg/kg/min during the infusion period. Similar to rs-fMRI acquisition, the following physiological parameters were monitored and recorded electronically twice: once before MRI Scan1 (without dobutamine infusion) and another before MRI Scan2 (with dobutamine infusion) using patient monitor (BeneVision N12, Mindray Medical International Ltd., Shenzhen, China): (1) heart rate (HR), (2) SpO2, (3) respiration rate, (4) blood pressure (BP), and (5) ECG. All physiological data, except BP, which was sampled every 2 min, were acquired at 1 Hz. The values of vital signs parameters in the participant’s steady state were obtained by averaging the values over the monitoring time period.

All participants underwent cognitive assessment on the day of scanning. The initial screening of cognitive function was conducted using the Montreal Cognitive Assessment (MoCA) battery, which consisted of five dimensions, including visuospatial functions, executive functions, language, attention, and memory. To assess subjective changes in the perception of dobutamine infusion, the participants were asked to select the description that best matched their current state on a visual analog scale (VAS) from 1 to 5 for “no sensation,” “mild,” “moderate,” “strong,” and “overwhelming,” respectively. The MoCA and VAS tests were performed twice in the same order for each participant, once before the normal rs-fMRI scan (Scan1) and once after the dobutamine infusion rs-fMRI scan (Scan2), to provide a complete profile of the participants’ cognitive function. Forty-eight healthy males (*n* = 43) and females (*n* = 5) between the ages of 23 and 34 were included for subsequent analysis after quality control. Four participants failed to complete all examinations, including one participant who was found to have adverse signs during ECG monitoring prior to Scan 1, three who failed to complete Scan 2 due to adverse reactions during dobutamine infusion.

### 2.3. Image preprocessing

Magnetic resonance imaging image data preprocessing was performed using data processing and analysis of brain imaging (DPABI) v6.0 toolbox in MATLAB.^1^ The first 10 volumes were discarded to allow the signal to stabilize. Slice-timing correction and motion realignment were conducted for the remaining 190 volumes. Nuisance parameters, including Friston 24 head-motion parameters, white matter signal, cerebrospinal fluid signal, and linear trend, were regressed out. The derived functional images were spatially normalized to the Montreal Neurological Institute (MNI) coordinate space with a voxel size of 3 × 3 × 3 mm and band-pass filtered (0.01–0.1 Hz). Fisher’s r-to-z transformation was utilized to improve the overall normality of the correlations before subsequent statistical analysis.

DPABI v6.0 was utilized for ReHo and ALFF analyses. ReHo method was used to analyze the local synchronization of brain activity in all participants, and was calculated by reducing low-frequency drift and high frequency noise, both of which were performed by passing the spatially standardized data through a 0.01–0.1 Hz bandpass filter. The similarity between a single voxel and its surrounding 26 voxels was calculated using Kendall’s coefficient of concordance (KCC) (Zang et al., 2004), which was then converted to a Z score by subtracting the mean value of the whole brain and dividing it by the standard deviation. Finally, the ReHo brain map was smoothed using a 6 × 6 × 6 full-width at half maximum (FWHM) kernel.

The amplitude of low-frequency fluctuation (ALFF) was calculated to indicate the regional intensity of spontaneous brain activity. The time series of each voxel was transformed into the frequency domain using the fast Fourier transform algorithm to obtain the power spectrum. The square root was then calculated at each frequency of the power spectrum and averaged across 0.01–0.1 Hz at each voxel. This averaged square root was considered the ALFF (Yan et al., 2013). The global mean was subtracted from the ALFF of each voxel and divided by the whole-brain standard deviation to yield a standardized Z score. Finally, the Z maps were smoothed with a 6 mm FWHM kernel.

### 2.4. Statistical analysis

Before-after-infusion comparisons of physiological parameters and cognitive assessment were analyzed using paired *t*-test under GraphPad Prism (Version 9.1.1, GraphPad Software, LLC, San Diego, CA, USA). Whole-brain ReHo and ALFF maps were compared using paired *t*-test in the statistical analysis module of DPABI v6.0 and Gaussian random field (GRF) corrected (voxelwise *p*-value < 0.001, cluster *p*-value < 0.01, two-tailed) (Worsley et al., 1996). Significant clusters were identified as regions of interest (ROI) and the standardized ReHo and ALFF values of these ROIs were extracted. The effects of physiological features on the changes in spontaneous brain activity were analyzed subsequently. Five physiological parameters, including age, BMI, and before-after-infusion differences in heart rate, systolic blood pressure, and diastolic blood pressure, were introduced as independent variables in a logistic regression analysis, with the difference in ReHo/ALFF in each ROI as the dependent variable. If the ReHo/ALFF change was greater than the mean of absolute values, it was positive sample; otherwise, it was negative sample. Regression analysis was performed in SPSS 26.0. A significance level was set at *p* < 0.05.

## 3. Results

### 3.1. Participant characteristics

General characteristics of the participants before and after dobutamine infusion are shown in Table 1. After dobutamine infusion, significant increases were observed in basic physiological parameters, including heart rate, blood pressure (SBP, DBP, and MABP) (all *p* < 0.0001), and respiration rate (*p* = 0.002). However, the cognitive assessments did not differ significantly before and after the infusion (MoCA score, *p* = 0.382; VAS score, *p* = 0.083).

**TABLE 1:** General characteristics of participants (*N* = 48).

Sample characteristics	Before infusion	After infusion	*P*-value
**Demographics**			
Sex, *N* (male/female)	48 (43/5)		
Age, years, mean (SD) [range]	26.33 (3.19) [23–34]		
BMI, mean (SD) [range]	22.78 (2.59) [18–31]		
Heart rate, BPM, mean ± SD	68.21 @11.76	84.83 @18.00	<0.0001
**Blood pressure**			
SBP, mmHg, mean ± SD	118.81 @9.97	158.56 @12.65	<0.0001
DBP, mmHg, mean ± SD	73.88 @7.96	83.67 @7.77	<0.0001
MABP, mmHg, mean ± SD	88.85 @8.18	108.63 @8.17	<0.0001
Respiration rate, RPM, mean ± SD	17.29 @3.88	18.25 @4.05	0.002
SpO2,%, mean ± SD	98.85 @1.11	98.94 @1.04	0.420
**Cognitive assessment**			
MoCA score, mean ± SD	27.57 @1.95	27.88 @1.61	0.382
VAS score, mean ± SD	1.06 @0.24	1.13 @0.33	0.083

BMI, body mass index; BPM, beats per minute; SBP, systolic blood pressure; DBP, diastolic blood pressure; MABP, mean arterial blood pressure; RPM, rate per minute, MoCA, Montreal Cognitive Assessment; VAS, visual analog scale. Physiological parameters and cognitive assessment were analyzed using paired *t*-test. A significance level was set at *p* < 0.05.

### 3.2. Alterations of ReHo and ALFF in dobutamine infusion states

**FIGURE 2:**
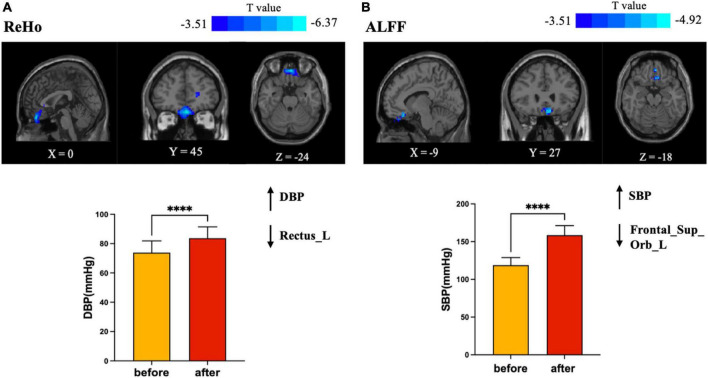
Brain regions in which brain functional activities associated with blood pressure. **(A)** Plots of elevated DBP with a decrease in regional homogeneity (ReHo) of the left gyrus rectus; **(B)** plots of elevated SBP with a decrease in amplitude of low frequency fluctuation (ALFF) of the left frontal superior orbital gyrus. SBP changed more dramatically than DBP, but ALFF decreased less than ReHo (*T* value = –4.92 versus *T* value = –6.37). SBP, systolic blood pressure; DBP, diastolic blood pressure. ****Statistical different results with *P* < 0.0001.

Analysis of the ReHo and ALFF maps revealed significant reductions in spontaneous neural activity. Compared with the normal resting state without dobutamine infusion, decreases in both ReHo and ALFF were found in the frontal lobe in the dobutamine infusion state (see Table 2). Decreases in ReHo were mainly observed in the left gyrus rectus, while decreases in ALFF were found in the left frontal superior orbital (GRF-corrected, cluster *p* < 0.01, voxelwise *p* < 0.001) (see Figure 2).

**TABLE 2:** Brain regions showing altered ReHo and ALFF in dobutamine infusion states.

Brain regions	Cluster size	MNI			*T*-value
		x	y	z	
**ReHo**					
Rectus_L	359	0	45	−24	−6.37
**ALFF**					
Frontal_Sup_Orb_L	109	−9	27	−18	−4.92

L, left; MNI, Montreal Neurological Institute; ReHo, regional homogeneity; ALFF, amplitude of low frequency fluctuation.

### 3.3. Associations between the altered brain regions and clinical data

The logistic regression model showed that the before-after-infusion difference in diastolic blood pressure was associated with the change in the ReHo value of the left gyrus rectus [*B* = 1.813, odds ratio (OR) = 6.130; 95% CI = (1.344, 27.950)] (see Table 3). The before-after-infusion difference in systolic blood pressure was associated with the change in the ALFF value of the left frontal superior orbital [*B* = −1.218, OR = 0.296; 95% CI = (0.099, 0.881)] (see Table 4 and Figure 2).

**TABLE 3:** Factors affecting the regional homogeneity (ReHo) value of the left gyrus rectus.

	B	S.E.	Wald	Sig.	OR	95% CI
Age	−1.282	0.787	2.656	0.103	0.277	0.059∼1.297
BMI	0.679	1.066	0.406	0.524	1.972	0.244∼15.937
HR_difference	−0.260	0.621	0.175	0.676	0.771	0.228∼2.606
SBP_difference	−0.216	0.617	0.122	0.726	0.806	0.240∼2.701
DBP_difference	1.813	0.774	5.486	0.019	6.130	1.344∼27.950

HR, heart rate; SBP, systolic blood pressure; DBP, diastolic blood pressure; B, regression coefficients; S.E., standard error; Wald, chi-square value, equal to (B/S.E.)^∧2^; Sig., significance level was set at *p* < 0.05; OR, odds ratio; CI, confidence interval.

**TABLE 4:** Factors affecting the amplitude of low frequency fluctuation (ALFF) value of the left frontal superior orbital.

	B	S.E.	Wald	Sig.	OR	95% CI
Age	0.406	0.667	0.371	0.542	1.501	0.406∼5.549
BMI	−0.324	0.826	0.154	0.695	0.723	0.143∼3.650
HR_difference	0.197	0.497	0.157	0.692	1.218	0.459∼3.228
SBP_difference	−1.218	0.557	4.782	0.029	0.296	0.099∼0.881
DBP_difference	0.651	0.549	1.403	0.236	1.917	0.653∼5.628

HR, heart rate; SBP, systolic blood pressure; DBP, diastolic blood pressure; B, regression coefficients; S.E., standard error; Wald, chi-square value, equal to (B/S.E.)^∧2^; Sig., significance level was set at *p* < 0.05; OR, odds ratio; CI, confidence interval.

## 4. Discussion

In this study, cardiac function was altered in healthy young adults by dobutamine infusion and the resulting changes in spontaneous brain activity were analyzed to investigate the brain-heart interactions. The results demonstrated that for healthy young adults, cognitive function was temporarily unaffected when heart rate and blood pressure increased after dobutamine infusion, but spontaneous neural activity in the brain was altered, as evidenced by a decrease in ReHo and ALFF in the frontal lobe. Moreover, the degree of altered spontaneous brain activity was related to changes in systolic and diastolic blood pressure.

To the best of our knowledge, this is the first study that investigates the brain-heart interactions in healthy young adults under dobutamine challenge. Dobutamine was chosen for this study because its cardiovascular effects include significant inotropic rather than chronotropic effects on the heart, resulting in increased cardiac output and elevated blood pressure, which can act as an acute stimulus affecting brain-heart interactions (Tang et al., 2021). Dobutamine has no direct effect on brain activity as studies have shown that peripherally infused dobutamine did not interact with brain β1 receptors or exert direct vasomotor effects on brain microvessels in rodents (Conway et al., 1987). Theoretically, the BOLD signal is related to cerebral blood flow (CBF), cerebral blood volume and blood oxygenation, while a study showed that dobutamine infusion did not increase internal carotid artery (ICA) blood flow despite increases in cardiac output and arterial blood pressure (Ogoh et al., 2017). Therefore, any changes in spontaneous neural activity would be interpreted as the result of changes in cardiovascular system function (heart rate, blood pressure, and other vital signs).

In this study, blood pressure and heart rate increased significantly, and the increase in SBP was more pronounced compared to DBP. This finding is consistent with the performance of dobutamine at higher infusion doses. Blood pressure is the pulsatile dilating pressure exerted on blood vessels throughout the body and is essential for maintaining oxygen delivery to body tissues. It is determined by the pumping action of the heart (cardiac output) and the contractile state of the smooth muscle of the small arteries, both of which are controlled by the autonomic nervous system (Kobuch et al., 2019). From a brain-heart interactions perspective, it has been suggested that both cortical and subcortical brain regions regulate blood pressure and other cardiovascular responses to behavioral stressors, including the mid-anterior, peripatellar, and infrapatellar regions of the cingulate cortex, the orbital and medial regions of the prefrontal cortex, the insula, and the hypothalamus, regulating neuroendocrine and autonomic nervous system control of the cardiovascular system (Gianaros et al., 2005). It has even been shown that even subtle differences in resting mean blood pressure, are associated with significant differences in resting activity in brain regions involved in the regulation of cardiovascular function (Kobuch et al., 2019). The results of the binary logistic regression in this study also showed that blood pressure was the main factor that influenced the alteration of spontaneous brain activity. The change of DBP had a significant positive relationship with the degree of change of ReHo [*B* = 1.813, odds ratio (OR) = 6.130; 95% CI = (1.344, 27.950)], and the change of SBP had a significant negative impact on the degree of change in ALFF [*B* = −1.218, OR = 0.296; 95% CI = (0.099, 0.881)]. That is, the more severe the before-after-infusion changes in DBP, the greater the difference in the ReHo of the left gyrus rectus, while the more severe before-after-infusion changes in SBP, the smaller the difference in ALFF of the left frontal superior orbital gyrus. These results may also be corroborated in the present study, that the change of SBP is more severe than that of DBP, but the degree of change of ReHo (cluster size = 109, *T* value = −4.92) was lower than that of ALFF (cluster size = 359, *T* value = −6.37). The elevated heart rate but reduced functional activity in the corresponding regions in the present study is also consistent with the results of a study linking regional BOLD fluctuations to heart rate, in that a negative correlation occurred between the bilateral orbitofrontal cortex and heart rate (Kassinopoulos et al., 2021).

The decreases in spontaneous brain activity were localized to the frontal lobe. The frontal lobe, located anterior to the central sulcus, is often considered the “choice center” that manages incoming information and selects appropriate actions based on motivation and goals. It is involved in the top-down control of cognition and behavior, often referred to as executive functions (Rosch and Mostofsky, 2019). Damage to this area can cause impairment of higher-order functions, such as voluntary movement, language expression, decision-making, planning, and memory (Rolls, 2019). However, no obvious impairment of language function was found in our study, as there was no significant difference in MoCA scores before and after dobutamine infusion. This result indicates that the frontal lobe neural activities change greatly in healthy young people during acute cardiovascular system function changes. Although no significant effect on cognitive function has been found for the time being, there are still potential risks that need further attention. In addition to being implicated in some cognitive functions, the prefrontal lobe is also involved in the regulation of cardiovascular function, and more susceptible to the cardiac pulses (Tavares et al., 2009; Pereira et al., 2013; Raitamaa et al., 2021). The elevation of heart rate and blood pressure due to dobutamine infusion in this study, while the prefrontal lobes showing a decrease in brain functional activity (reduction in ALFF seen in the left frontal superior orbital region and the reduction in ReHo in the left gyrus rectus), may be a result of this function.

It is noteworthy that these changes in functional brain activity show hemispheric lateralization, as changes occurring only on the left side. For the prefrontal cortex, according to the right hypothesis of emotion, the left hemisphere dominates cognition, and according to the valence asymmetry hypothesis, positive emotions are lateralized toward the left and negative emotions toward the right hemisphere (Beraha et al., 2012; Güntürkün et al., 2020). Studies also have shown that the prefrontal cortex on the left is more susceptible to high corticosteroid levels and may be more susceptible to stress. Although the underlying mechanisms leading to this phenomenon remain unclear, this laterality of vulnerability to carbohydrate/stress may be a general phenomenon (Cerqueira et al., 2008). Furthermore, participants exposed to transient, behaviorally relevant cardiovascular challenges resulted in discrete changes in activity across multiple brain sites, and these activity changes were often highly lateralized in specific prefrontal and temporal forebrain regions (Harper et al., 2000). Due to the different stimulation methods used in this study and the functional localization of major changes in brain regions, the phenomenon of lateralization also needs to be further verified in subsequent studies in combination with emotional and cognitive assessments.

There are several limitations in this study. Firstly, the participants recruited in this study were predominantly males under 35 years, and there is still a need to include different age and gender groups in future studies for the comprehensiveness of the findings. Secondly, the indicators used to assess changes in cardiovascular function in the study were heart rate and blood pressure, and ejection fraction, heart rate variability, venous or even intracranial pressure will be used to explore the interactions between the two system. Thirdly, changes to the experimental protocol, such as using faster fMRI acquisitions, changing the sequence of scans or cognitive assessments, may be considered in subsequent studies to obtain more stable and reliable results. Because studies have shown that when a longer TR time is used, the physiological parameter signal may alias with the BOLD signal, which will have a certain impact on the analysis of frequency domain indicators, and a shorter TR time is more conducive to rs-fMRI analysis (Raitamaa et al., 2021; Huotari et al., 2022). Moreover, subjects may present different brain BOLD signals between awake and asleep, even in the resting state (Tagliazucchi and Laufs, 2014; Liu and Falahpour, 2020; Helakari et al., 2022). Therefore, state monitoring during rs-fmri scanning is also necessary. We will monitor them in subsequent studies using EEG or MRI multiparametric subject state monitoring system (MD-M100, Shenzhen Sinorad Medical Electronic Co., Ltd., Shenzhen, China) to ensure the stability of the acquired signal. Additionally, functional integration methods such as FC, ICA, and graph analysis will provide more stable results of the brain-heart interactions, as the smaller effect of sampling frequency on these metrics has been demonstrated in study (Huotari et al., 2019), which we will analyze in the future.

With the increased risk of cardiovascular disease transfer to the young people, it becomes particularly important to explore the corresponding neurological changes and to guide the protection of the brain-heart system. This study induced acute changes in cardiovascular system function by infusion of dobutamine and explored the corresponding changes in spontaneous brain activity. Our data showed changes in spontaneous neural activity in the frontal lobe after dobutamine infusion, including a ReHo decrease in the left gyrus rectus gyrus and an ALFF decrease in the left superior frontal gyrus. Blood pressure was the main factor that influenced the alteration of spontaneous brain activity in these regions. This suggests that more attention should be paid to the changes in the blood pressure of the young adults, and functional assessments for the frontal lobe may be performed in future to monitor the neural integrity of the young adults. This knowledge could contribute to more efficient risk-factor management in the younger population.

## Data availability statement

The raw data supporting the conclusions of this article will be made available from the corresponding authors upon request.

## Ethics statement

The studies involving human participants were reviewed and approved by Medical Research Ethics Committee and Institutional Review Board of Beijing Friendship Hospital, Capital Medical University. The patients/participants provided their written informed consent to participate in this study.

## Author contributions

YL, PR, and ZW planned the experiments. YL, PR, TZ, LC, DL, and LZ completed the data collection and conducted the experiments. YL, EZ, and PQ designed the methodology and analyzed the data. YL and PR wrote the initial draft. HN, WZ, and ZW supervised the entire experiment and revised the draft article. ZW acquired the financial support for the experiment. All authors approved the final version of the manuscript and agreed to accountable for all aspects of the work.

## Footnotes

1
http://rfmri.org/dpabi
